# Risk factors for hemorrhoidal disease among healthy young and middle-aged Korean adults

**DOI:** 10.1038/s41598-021-03838-z

**Published:** 2022-01-07

**Authors:** Yun Soo Hong, Kyung Uk Jung, Sanjay Rampal, Di Zhao, Eliseo Guallar, Seungho Ryu, Yoosoo Chang, Hyung Ook Kim, Hungdai Kim, Ho-Kyung Chun, Chong Il Sohn, Hocheol Shin, Juhee Cho

**Affiliations:** 1 Departments of Epidemiology and Medicine, and Welch Center for Prevention, Epidemiology, and Clinical Research, Johns Hopkins University Bloomberg School of Public Health, Baltimore, MD USA; 2 Department of Surgery, Kangbuk Samsung Hospital, Sungkyunkwan University School of Medicine, 29 Saemunan-ro, Jongno-gu, Seoul, 03181 South Korea; 3 Department of Social and Preventive Medicine, Julius Centre University of Malaya, Faculty of Medicine, University of Malaya, Kuala, Lumpur, Malaysia; 4 Department of Clinical Research Design & Evaluation, SAIHST, Sungkyunkwan University, 81 Irwon-ro, Gangnam-gu, Seoul, 06351 Republic of Korea; 5 Center for Cohort Studies, Total Healthcare Center, Kangbuk Samsung Hospital, Sungkyunkwan University School of Medicine, Seoul, Republic of Korea; 6 Department of Occupational and Environmental Medicine, Kangbuk Samsung Hospital, Sungkyunkwan University School of Medicine, Seoul, Republic of Korea; 7 Division of Gastroenterology, Department of Internal Medicine and Gastrointestinal Cancer Center, Kangbuk Samsung Hospital, Sungkyunkwan University School of Medicine, Seoul, Korea; 8 Department of Family Medicine, Kangbuk Samsung Hospital, Sungkyunkwan University School of Medicine, Seoul, South Korea; 9 Center for Clinical Epidemiology, Samsung Medical Center, Seoul, South Korea

**Keywords:** Public health, Anal diseases, Gastroenterology, Colonoscopy

## Abstract

Hemorrhoidal disease is a highly prevalent anorectal condition causing substantial discomfort, disability, and decreased quality of life. Evidence on preventable risk factors for hemorrhoidal disease is limited. We conducted a cross-sectional study of 194,620 healthy men and women who completed a health screening exam including colonoscopy in 2011–2017. We evaluated potential risk factors of hemorrhoidal disease, including lifestyle factors, medical history, birth history, gastrointestinal symptoms, and anthropometric measurements. The prevalence of hemorrhoidal disease was 16.6%, and it was higher in females than in males (17.2 vs. 16.3%; *P* < 0.001). Compared to men, the prevalence of hemorrhoidal disease was higher in parous women (adjusted odds ratio [OR] 1.06; 95% confidence interval [CI] 1.02–1.10), and lower in nulliparous women (adjusted OR 0.92; 95% CI 0.86–0.98). In the adjusted analyses, older age, female sex, smoking, overweight, and being hypertensive were independently associated with the presence of hemorrhoidal disease. The prevalence of hemorrhoidal disease was positively associated with body mass index and waist circumference in parous women. The prevalence of hemorrhoidal disease was higher in older age, females, ever-smokers, and hypertensive participants. The association of excess adiposity with the prevalence of hemorrhoidal disease differed by sex and parity.

## Introduction

Hemorrhoids are normal vascular cushions surrounding the distal rectum and anal canal that help maintain anal continence^1,2^. The term hemorrhoids, however, commonly refers to pathological changes and distal displacement of hemorrhoidal tissue (hemorrhoidal disease), affecting nearly 40% of adults^3,4^. Hemorrhoidal disease can cause substantial discomfort, disability, and a decrease in quality of life^5^. It is also a major medical and socioeconomic problem, and represents a major burden to the health care system^6,7^.

Management of hemorrhoidal disease ranges from noninvasive medical approaches to invasive surgical procedures based on its disease grade^8–14^. Noninvasive methods do not reverse the underlying structural changes in the hemorrhoidal tissue, while invasive procedures have a relatively high frequency of complications and recurrence. Moreover, the severity and symptoms of hemorrhoidal disease tend to aggravate over time if it is not managed properly. Thus, the alternative approach to hemorrhoidal disease should be based on active prevention and early intervention, including dietary changes, medical treatment, and modification of potential risk factors^15^.

The evidence base on preventable risk factors for hemorrhoidal disease, however, is limited. Older age, female gender, lower socioeconomic status, lack of physical activity, pregnancy, alcohol consumption, constipation, diarrhea, prolonged abdominal straining, sedentary lifestyle, and obesity have been proposed as risk factors, but the findings are inconsistent across studies^3,4,6,16–18^. In addition, although previous studies have evaluated the associaiton of body mass index (BMI) with hemorrhoidal disease, the association of different measures of adiposity, including body fat distribution (central obesity) and body fat mass, and hemorrhoidal disease have not been explored. The objective of our study was thus to evaluate the risk factors associated with prevalent hemorrhoidal disease in a large study of healthy adults who underwent a health screening examination.

## Results

The mean (SD) age of study participants was 42.2 (9.4) years. Men were younger, more physically active, more likely to be smokers, and more likely to have higher education levels, alcohol intake, BMI, and waist circumference, lower % fat mass, and higher prevalence of diabetes, hypertension, and dyslipidemia compared to women (Table 1).

**Table 1:** Characteristics of study participants.

	Overall, *n* (%)	Sex, *n* (%)		*P*
	(*n* = 194,620)	Women (*n* = 69,185)	Men (*n* = 125,435)	
**Age**				< 0.001
20 to 29	11,474 (5.9)	3,679 (5.3)	7,795 (6.2)	
30 to 39	80,366 (41.3)	26,840 (38.8)	53,526 (42.7)	
40 to 49	63,853 (32.8)	22,305 (32.2)	41,548 (33.1)	
50 to 59	27,776 (14.3)	11,187 (16.2)	16,589 (13.2)	
60 to 70	11,151 (5.7)	5,174 (7.5)	5,977 (4.8)	
Education > 12 years	145,517 (74.8)	44,850 (64.8)	100,667 (80.3)	< 0.001
**Physical activity**				< 0.001
Low	92,330 (47.4)	37,063 (53.6)	55,267 (44.1)	
Moderate	67,163 (34.5)	20,445 (29.6)	46,718 (37.2)	
High	32,736 (16.8)	10,503 (15.2)	22,233 (17.7)	
**Smoking**				< 0.001
Never	87,113 (44.8)	55,494 (80.2)	31,619 (25.2)	
Former	53,466 (27.5)	4,642 (6.7)	48,824 (38.9)	
Current	42,379 (21.8)	1,583 (2.3)	40,796 (32.5)	
**Daily alcohol intake**				< 0.001
None	20,447 (10.5)	13,953 (20.2)	6,494 (5.2)	
< 5 g/day	56,053 (28.8)	29,793 (43.1)	26,260 (20.9)	
5 to < 20 g/day	60,666 (31.2)	13,538 (19.6)	47,128 (37.6)	
≥ 20 g/day	45,816 (23.5)	3,680 (5.3)	42,136 (33.6)	
Body mass index (kg/m^2^)	23.8 (3.3)	22.2 (3.2)	24.7 (3.0)	< 0.001
% Fat mass (%)	26.0 (6.6)	30.6 (6.1)	23.4 (5.4)	< 0.001
Waist circumference (cm)	83.1 (9.4)	76.9 (8.5)	86.6 (8.0)	< 0.001
Central obesity (%)	51,425 (26.4)	11,444 (16.5)	39,981 (31.9)	< 0.001
Diabetes	10,254 (5.3)	2,429 (3.5)	7,825 (6.2)	< 0.001
Hypertension	27,826 (14.3)	6,099 (8.8)	21,727 (17.3)	< 0.001
Dyslipidemia	8,604 (4.4)	2,943 (4.3)	5,661 (4.5)	0.001

Values in the Table are number (%) or mean (SD).

The prevalence of hemorrhoidal disease was 16.6% (*n* = 32,347; Table 2), and it was higher in females than in males (17.2 vs. 16.3%; *P* < 0.001). Compared to men, parous women had a higher prevalence of hemorrhoidal disease (adjusted OR 1.06; 95% CI 1.02–1.10), but nulliparous women had a lower prevalence than men (adjusted OR 0.92; 95% CI 0.86–0.98). Among parous women, the prevalence of hemorrhoidal disease did not differ by type of birth (adjusted OR for ever given natural birth vs. cesarean section only 0.97; 95% CI 0.91–1.04) or by number of births (adjusted ORs for 2 and 3 or more births vs. 1 birth 0.98; 95% CI 0.93–1.04 and 0.97; 95% CI 0.90–1.05, respectively).

**Table 2:** Association between participant characteristics and the prevalence of hemorrhoidal disease.

	Hemorrhoidal disease, *n* (%)	Crude odds ratio, OR (95% CI)	*P* for trend	Adjusted odds ratio*, OR (95% CI)	*P* for trend
Overall	32,347 (16.6)				
**Age**			< 0.001		< 0.001
20 to 29	1,386 (12.1)	1.00 (ref)		1.00 (ref)	
30 to 39	11,152 (13.9)	1.17 (1.10, 1.24)		1.23 (1.16, 1.31)	
40 to 49	11,444 (17.9)	1.59 (1.50, 1.69)		1.62 (1.53, 1.72)	
50 to 59	5,908 (21.3)	1.97 (1.85, 2.09)		1.99 (1.87, 2.13)	
60 to 70	2,457 (22.0)	2.06 (1.91, 2.21)		2.06 (1.91, 2.22)	
**Sex**			< 0.001		0.002
Men	20,458 (16.3)	1.00 (ref)		1.00 (ref)	
Women	11,889 (17.2)	1.06 (1.04, 1.09)		1.06 (1.02, 1.09)	
**Sex / parity**			< 0.001		0.002
Men	20,458 (16.3)	1.00 (ref)		1.00 (ref)	
Women, nulliparous	1,432 (13.5)	0.80 (0.76, 0.85)		0.92 (0.86, 0.98)	
Women, parous	9,484 (17.7)	1.10 (1.07, 1.13)		1.06 (1.02, 1.10)	
Natural birth, ever^†^	7,001 (17.7)	1.10 (1.07, 1.14)		1.04 (1.00, 1.08)	
C-section only^†^	1,297 (18.3)	1.15 (1.08, 1.23)		1.08 (1.01, 1.16)	
**Education**			< 0.001		0.75
> 12 years	23,598 (16.2)	1.00 (ref)		1.00 (ref)	
≤ 12 years	6,752 (18.4)	1.16 (1.13, 1.20)		1.01 (0.97, 1.04)	
**Physical activity**			0.30		0.78
Low	15,393 (16.7)	1.00 (ref)		1.00 (ref)	
Moderate	10,904 (16.2)	0.97 (0.94, 1.00)		0.97 (0.94, 0.99)	
High	5,624 (17.2)	1.04 (1.00, 1.07)		0.99 (0.96, 1.03)	
**Smoking status**			0.60		0.28
Never	14,309 (16.4)	1.00 (ref)		1.00 (ref)	
Former	9,083 (17.0)	1.04 (1.01, 1.07)		1.05 (1.01, 1.09)	
Current	6,956 (16.4)	1.00 (0.97, 1.03)		1.06 (1.02, 1.10)	
**Daily alcohol intake**			0.13		0.34
None	3,530 (17.3)	1.00 (ref)		1.00 (ref)	
< 5 g/day	9,267 (16.5)	0.95 (0.91, 0.99)		1.01 (0.97, 1.06)	
5 to < 20 g/day	9,854 (16.2)	0.93 (0.89, 0.97)		1.02 (0.97, 1.06)	
≥ 20 g/day	7,632 (16.7)	0.96 (0.92, 1.00)		1.01 (0.97, 1.07)	
**Body mass index**			0.001		0.75
Underweight	1,047 (15.2)	0.92 (0.86, 0.99)		0.98 (0.92, 1.05)	
Normal	12,303 (16.3)	1.00 (ref)		1.00 (ref)	
Overweight	8,318 (17.3)	1.07 (1.04, 1.11)		1.04 (1.01, 1.08)	
Obese	10,679 (16.7)	1.03 (1.00, 1.06)		1.00 (0.97, 1.03)	
**Diabetes**			< 0.001		0.07
No	30,411 (16.5)	1.00 (ref)		1.00 (ref)	
Yes	1,918 (18.7)	1.16 (1.11, 1.23)		0.96 (0.92, 1.00)	
**Hypertension**			< 0.001		0.001
No	27,041 (16.2)	1.00 (ref)		1.00 (ref)	
Yes	5,244 (18.9)	1.20 (1.16, 1.24)		1.04 (1.02, 1.07)	
**Dyslipidemia**			< 0.001		0.93
No	30,608 (16.5)	1.00 (ref)		1.00 (ref)	
Yes	1,724 (20.0)	1.27 (1.20, 1.34)		1.00 (0.95, 1.05)	

* Adjusted for age (20–29, 30–39, 40–49, 50–59, and 60–70 years), sex, year of visit, center, education (≤ 12 and > 12 years), physical activity (low, moderate, and high), smoking (never, former, and current), daily alcohol intake (none, < 5 g/day, 5 to < 20 g/day, and ≥ 20 g/day), BMI category (underweight, normal, overweight, and obese), and presence of diabetes, hypertension, and dyslipidemia. The ORs for sex / parity categories were obtained from a model with the same covariates but replacing sex with sex / parity categories.

^†^ Excluding participants without information on the mode of delivery.

The prevalence of hemorrhoidal disease was higher in older participants, in those with lower education level, in those who were overweight or obese, or former smokers, and in those with diabetes, hypertension, or dyslipidemia. In adjusted analyses, increasing age, female sex, former or current smoking, being overweight, and being hypertensive were independently associated with the prevalence of hemorrhoidal disease (Table 2, Appendix Table 1). In analyses performed separately by sex and parity (Appendix Table 2), age and smoking were stronger risk factors in men than in nulliparous women and in parous women (*P* interaction < 0.001 and 0.30, respectively). In addition, being overweight or obese was a particularly strong risk factor in parous women (*P* interaction 0.07), and having dyslipidemia was the strongest risk factor in nulliparous women (*P* interaction 0.007).

When we studied measures of adiposity other than BMI (Table 3), % fat mass showed an inverse association (*P* for trend < 0.001) with the prevalence of hemorrhoidal disease in fully adjusted models, while there was no clear trend with waist circumference and central obesity. In sex and parity specific models (Appendix Table 3), there was an inverse association of % fat mass with the prevalence of hemorrhoidal disease was inverse in men and nulliparous women (*P* interaction 0.46), and a positive association of waist circumference in parous women (*P* interaction < 0.001). Central adiposity showed an inverse association with the prevalence of hemorrhoidal disease in men (adjusted OR 0.96; 95% CI 0.93–1.00), and a positive association in parous women (adjusted OR 1.08; 95% CI 1.02–1.15).

**Table 3:** Association between alternative markers of adiposity and the prevalence of hemorrhoidal disease.

	Hemorrhoids, *n* (%)	Crude Odds Ratio, OR (95% CI)	*P* for trend	Adjusted Odds Ratio*, OR (95% CI)	*P* for trend
**% Fat mass**			< 0.001		< 0.001
Quintile 1	6,184 (15.9)	1.00 (ref)		1.00 (ref)	
Quintile 2	6,419 (16.5)	1.04 (1.01, 1.09)		0.99 (0.95, 1.02)	
Quintile 3	6,432 (16.5)	1.05 (1.01, 1.09)		0.96 (0.93, 1.00)	
Quintile 4	6,556 (16.8)	1.07 (1.03, 1.11)		0.96 (0.92, 1.00)	
Quintile 5	6,756 (17.4)	1.11 (1.07, 1.15)		0.92 (0.88, 0.96)	
**Waist circumference**			0.26		0.84
Quintile 1	6,098 (15.9)	1.00 (ref)		1.00 (ref)	
Quintile 2	6,606 (16.9)	1.07 (1.03, 1.11)		1.06 (1.02, 1.10)	
Quintile 3	6,681 (17.2)	1.09 (1.05, 1.13)		1.07 (1.02, 1.11)	
Quintile 4	6,607 (16.8)	1.07 (1.03, 1.11)		1.05 (1.00, 1.09)	
Quintile 5	6,355 (16.3)	1.03 (0.99, 1.07)		1.02 (0.97, 1.07)	
**Central obesity**			0.40		0.49
No	23,739 (16.6)	1.00 (ref)		1.00 (ref)	
Yes	8,608 (16.7)	1.01 (0.98, 1.04)		0.99 (0.96, 1.02)	

* Adjusted models included age (20–29, 30–39, 40–49, 50–59, and 60–70 years), sex, year of visit, center, education (≤ 12 and > 12 years), physical activity (low, moderate, and high), smoking (never, former, and current), daily alcohol intake (none, < 5 g/day, 5 to < 20 g/day, and ≥ 20 g/day), and presence of diabetes, hypertension, and dyslipidemia, plus one of the following markers of excess adiposity: % fat mass % categorized in quintiles (< 20.34, 20.35–23.75, 23.75–27.08, 27.08–31.58, ≥ 31.58%), waist circumference categorized in quintiles (< 74.7, 74.7–80.5, 80.5–85.1, 85.2–90.5, and ≥ 90.5 cm), and central obesity (no vs. yes).

Among self-reported digestive and lower gastrointestinal symptoms (Table 4), the presence of red blood in stools (OR 1.54; 95% CI 1.46–1.63), constipation (OR 1.10; 95% CI 1.06–1.14), narrow caliber stools (OR 1.05; 95% CI 1.01–1.10), and tenesmus (OR 1.05, 95% CI 1.02–1.09) were significantly associated with an increased prevalence of hemorrhoidal disease, while difficulty in digesting food (OR 0.96; 95% CI 0.93–0.99) was associated with a lower prevalence of hemorrhoidal disease. The associations between symptoms and the prevalence of hemorrhoidal disease were similar across sex and history of parity groups (Appendix Table 4).

**Table 4:** Association between self-reported gastrointestinal symptoms and the prevalence of hemorrhoidal disease.

	Hemorrhoids, *n* (%)	Crude Odds Ratio, OR (95% CI)	*P*	Adjusted Odds Ratio*, OR (95% CI)	*P*
Difficulty swallowing	184 (16.2)	0.97 (0.83, 1.14)	0.73	0.92 (0.78, 1.07)	0.28
Acid regurgitation	3,110 (17.1)	1.04 (1.00, 1.08)	0.05	1.01 (0.97, 1.05)	0.69
Nausea and/or vomiting	1,055 (15.5)	0.92 (0.86, 0.98)	0.01	0.96 (0.90, 1.03)	0.29
Difficulty in digestion	6,150 (15.8)	0.93 (0.90, 0.95)	< 0.001	0.96 (0.93, 0.99)	0.01
Heartburn	4,270 (16.6)	1.00 (0.96, 1.04)	0.97	0.98 (0.95, 1.02)	0.40
Tarry stools	690 (16.2)	0.97 (0.89, 1.05)	0.47	0.97 (0.90, 1.06)	0.53
Fresh blood in stools	1,820 (21.8)	1.42 (1.35, 1.50)	< 0.001	1.54 (1.46, 1.63)	< 0.001
Frequent diarrhea	4,324 (15.6)	0.92 (0.88, 0.95)	< 0.001	0.97 (0.93, 1.00)	0.06
Constipation	3,866 (17.8)	1.09 (1.05, 1.14)	< 0.001	1.10 (1.06, 1.14)	< 0.001
Narrow caliber stools	2,554 (17.6)	1.08 (1.03, 1.13)	0.001	1.05 (1.01, 1.10)	0.02
Lump or mass in the abdomen	498 (16.5)	0.99 (0.90, 1.10)	0.92	0.99 (0.90, 1.09)	0.87
Frequent abdominal pain	2,093 (15.9)	0.94 (0.90, 0.99)	0.02	1.01 (0.96, 1.06)	0.72
Tenesmus	5,269 (17.1)	1.04 (1.01, 1.07)	0.02	1.05 (1.02, 1.09)	0.004

* Adjusted for age (20–29, 30–39, 40–49, 50–59, and 60–70 years), sex, year of visit, center, education (≤ 12 and > 12 years), physical activity (low, moderate, and high), smoking (never, former, and current), daily alcohol intake (none, < 5 g/day, 5 to < 20 g/day, and ≥ 20 g/day), BMI category (underweight, normal, overweight, and obese), and presence of diabetes, hypertension, and dyslipidemia.

## Discussion

In this study of healthy Korean adults participating in a health screening program with a complete colonoscopic examination, increasing age, female sex, smoking, presence of hypertension, history of childbirth, and presence of fresh blood in stools, constipation, narrow caliber stool, or tenesmus were associated with hemorrhoidal disease. The association between adiposity and the prevalence of hemorrhoidal disease was complex and differed by sex and parity.

The overall prevalence of hemorrhoidal disease in our study was 16.6%. The prevalence of hemorrhoidal disease ranged from 4.4 to 88%^19–21^, depending on the study population and the methods of ascertainment (self-report of symptoms vs. physician diagnosis), but even recent studies using screening colonoscopy reported a wide range of prevalences (20.8 to 38.2%)^3,6,22^.

In our study, the prevalence of hemorrhoidal disease increased progressively with age, with the highest prevalence observed in the oldest age group. Some studies reported the highest prevalence of hemorrhoid among middle aged men and women, but these estimates were based on a smaller sample size and self-reports or physician diagnosis of hemorrhoidal disease^3^, which may be subject to misclassification^16^.

Parous women had a significantly higher prevalence of hemorrhoidal disease compared to men and to nulliparous women, likely due to mechanical changes of the pelvic floor because of increased intra-abdominal pressure, pelvic venous congestion, and damage during labor, and to hormonal factors affecting gastrointestinal motility during pregnancy^23^. In previous studies, the prevalence of hemorrhoidal disease was highest in the 2nd and 3rd trimester of pregnancy and the incidence was ~ 24% during the first 6 months of delivery^23–26^. Consistent with previous studies^3,6,23,24^, the number of childbirths was not associated with increased risk of hemorrhoidal disease in our study.

Smoking was also associated with an increased prevalence of hemorrhoidal disease, possibly by promoting systemic inflammation and by its effects on collagen metabolism^16,27–31^. Although, the association between hypertension and hemorrhoidal disease has not been described in other studies, hypertension facilitates vascular injury through oxidative stress and inflammation, and may, thereby, further damage adjacent connective tissue^32^.

The association of excess adiposity with hemorrhoidal disease is controversial^6,16^. In our study, the risk of hemorrhoidal disease did not increase linearly with increasing BMI, but overweight women had an increased prevalence of hemorrhoidal disease. In addition, % fat mass was inversely associated with prevalence of hemorrhoidal disease in men and nulliparous women, while waist circumference was associated with a higher prevalence of hemorrhoidal disease only in parous women. The association of excess adiposity and its distribution with the risk of hemorrhoidal disease is thus complex, and may depend on sex, parity, and other characteristics. Additional research is needed to confirm our findings in other populations.

Among various self-reported gastrointestinal symptoms, fresh blood in stools, constipation, narrow caliber stools, and tenesmus were associated with the presence of hemorrhoidal disease. These symptoms may be a consequence of hemorrhoidal disease and not an antecedent factor. Constipation can cause hemorrhoidal disease by increasing intra-abdominal pressure, resulting in hemorrhoidal plexus engorgement, and in excessive straining during defecation leading to shearing forces on the anal cushions^3,6,16,33^.

There are several limitations to our study. As a cross-sectional study, we cannot assume temporality or causality between the risk factors and hemorrhoidal disease. Although a detailed examination of the anal and rectal areas is the standard-of-care in colonoscopy, the severity of hemorrhoidal disease, such as the Goligher classification, was not collected. Moreover, as the study population was participants undergoing a routine screening exam and not an evaluation for hemorrhoidal disease, an anoscopy was not performed in addition to a routine colonoscopy. In addition, bowel preparation prior to colonoscopy may increase congestion of the internal hemorrhoidal plexus and result in over-diagnosis or over-staging. Furthermore, the definition of hemorrhoidal disease in our study included any internal and/or external hemorrhoidal disease assessed by colonoscopy and may have included enlargement/bleeding of external hemorrhoidal plexus, which is considered to have a different pathology. The heterogeneity in the definition of hemorrhoidal disease around internal and external hemorrhoidal disease in the literature and in clinical practice may also lead to inconsistent results across studies. Moreover, because the questionnaire in this study was not designed specifically to screen for hemorrhoidal disease or to assess it severity, we were also unable to evaluate the severity of hemorrhoid-related anorectal symptoms. However, some of the anorectal symptoms included in the questionnaire, such as fresh blood in stools, constipation, and narrow caliber stools, were highly associated with the presence of hemorrhoidal disease.

In addition, study participants were predominantly young and middle-aged apparently healthy Korean adults without any previous history of colorectal disease. Therefore, the associations observed may not be generalized to other populations and race / ethnicity groups who have different genetic or lifestyle factors that might influence the presence of hemorrhoidal disease, or to populations with previous colorectal conditions.

Our study also has major strengths. We used a well-characterized cohort with high-quality information on multiple potential risk factors. In addition, this is the largest study to date evaluating risk factors for hemorrhoidal disease using a complete colonoscopic examination to ascertain the presence of disease. This approach is less likely to miss participants with asymptomatic hemorrhoidal disease and participants who might hide the disease due to embarrassment, which are major limitations of using claims-based data and self-reported questionnaire to define the presence of hemorrhoidal disease.

In this study, we found that the prevalence of hemorrhoidal disease increased with age and it was higher in parous women compared to men and to nulliparous women. Participants who ever smoked, who were hypertensive, and who reported fresh blood in stools or constipation also had an increased prevalence of hemorrhoidal disease. The association of excess adiposity with the prevalence of hemorrhoidal disease was complex and differed by sex and parity. Further epidemiological studies with longitudinal design are needed to better understand how these factors interact in the development of hemorrhoidal disease.

## Methods

### Study population

The Kangbuk Samsung Health Study is a cohort of adult men and women who underwent a comprehensive annual or biennial health screening exam at the Kangbuk Samsung Hospital Total Healthcare Screening Centers in Seoul and Suwon, South Korea^34,35^. Korean government mandates that employers provide regular health screening exams to their employees free of charge by the Industrial Safety and Health Law. Over 80% of the study participants in the Kangbuk Samsung Health Study are employees (or their relatives) of companies that contracted the routine health screening exams with the Kangbuk Samsung Hospital. The rest of the participants voluntarily purchased the health screening exams. For this study, we included participants 20 to 70 years of age who completed a health exam and had undergone at least one colonoscopy as part of routine health screening between April 2011 and December 2017, when the colonoscopy data in the Kangbuk Samsung Health Study was available (*n* = 212,517).

**Figure 1:**
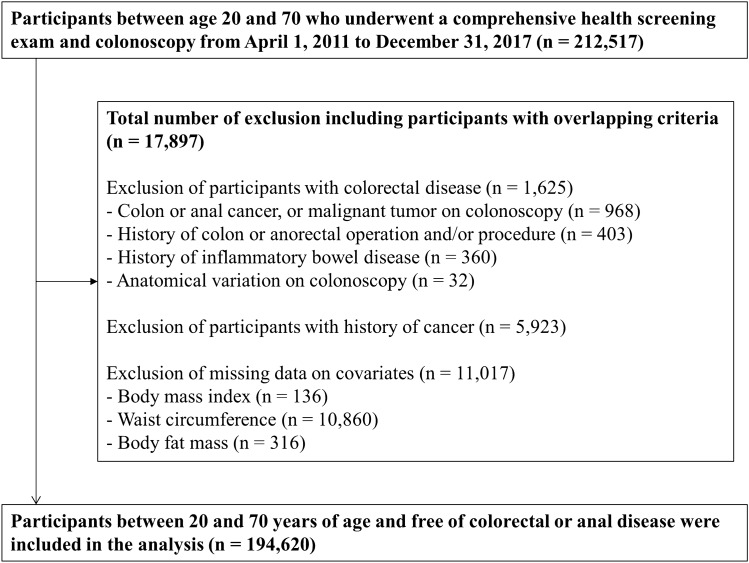
Flowchart of study participants.

We excluded participants with colorectal disease (*n* = 1,625), including colorectal or anal cancer findings on colonoscopy (*n* = 968), a history of colorectal or anorectal cancer operation and/or procedure (*n* = 403), a history of inflammatory bowel disease (*n* = 360), and anatomical variation on colonoscopy (*n* = 32); participants with a history of cancer (*n* = 5,923); and participants with missing data on BMI, waist circumference, or body fat mass (*n* = 11,017). The final sample size was 194,620 participants (125,4365 men and 69,185 women; Fig. 1). For participants who attended more than one screening exam during the study period, we included only data from the initial exam in the analysis.

The study was approved by the Institutional Review Board of the Kangbuk Samsung Hospital, which waived the requirement for informed consent because we used only de-identified information obtained as part of routine health screening exams. All research methods were performed in accordance with the relevant guidelines and regulations.

### Measurements

In each screening exam, study participants provided information on their medical history, smoking habits, alcohol consumption, physical activity, and education level through a standardized self-administered questionnaire. Smoking habits were categorized as never, former, or current. Alcohol consumption was calculated in g/day and further categorized into none, < 5 g/day, 5–20 g/day, and ≥ 20 g/day. We used the validated Korean version of the International Physical Activity Questionnaire Short Form (IPAQ-SF) to assess the frequency and duration of walking, moderate-intensity, and vigorous-intensity physical activity for more than 10 consecutive minutes across all daily activities over a 7-day span^36^. We then calculated an activity score as the number of minutes spent on each type of physical activity per week multiplied by the metabolic equivalent (MET) of each activity and categorized the level of physical activity into low, moderate, and high^37^. Education level was defined as ≤ 12 or > 12 years of education.

Height, weight, waist circumference, and body composition were measured by trained staff members. BMI was calculated as weight in kilograms divided by height in meters squared (kg/m^2^). BMI was classified according to the criteria proposed for Asian populations by the World Health Organization:^38^ underweight 15.0–18.4 kg/m^2^; normal 18.5–22.9 kg/m^2^; overweight 23.0–24.9 kg/m^2^; obese ≥ 25.0 kg/m^2^. Waist circumference was measured at the midpoint between the bottom of the rib cage and the top of the iliac crest to the nearest 0.1 cm in a standing position with weight equally distributed on both feet, arms at their sides, and head facing forward. Central obesity was defined by the guidelines suggested for Koreans (≥ 90 cm in men and ≥ 85 cm in women)^39^. % fat mass was measured using a multi-frequency bioimpedance analyzer (Inbody 3.0 and Inbody 720, Biospace Co., Seoul, Republic of Korea). Hypertension was defined as a systolic blood pressure ≥ 140 mmHg, a diastolic blood pressure ≥ 90 mmHg, a self-reported physician diagnosis, or current use of antihypertensive medication.

Clinical chemistry measurements were performed in venous blood samples obtained after more than 10 h of fasting. Diabetes was defined as a fasting serum glucose ≥ 126 mg/dl, a self-reported physician diagnosis, or current use of insulin or other hypoglycemic agents. Dyslipidemia was defined as a self-reported physician diagnosis, or current use of lipid lowering medication.

Prior to colonoscopy, participants were asked for the presence of any digestive or lower gastrointestinal symptoms from a list of 13 symptoms which were created based on clinical significance and frequency of report in previous health surveys at the center. The list included nausea and/or vomiting, acid regurgitation, heartburn, difficulty in swallowing, difficulty in digesting, frequent abdominal pain, constipation, frequent diarrhea, narrow-caliber stools, fresh blood in stools, tarry stools, tenesmus, and lumps or masses in the abdomen. For female participants, we also assessed the history of ever being pregnant, number of childbirths (parity), and mode of delivery for each birth (natural birth or cesarean section).

The presence of hemorrhoidal disease was assessed during colonoscopy performed as part of routine screening exams. Although colonoscopy was used to diagnose the presence of hemorrhoidal disease, the purpose of the exam was to screen for colorectal neoplasms and/or other colorectal conditions. Rectal examination was performed prior to insertion of the colonoscope. Colonoscopies was performed by experienced gastroenterologists using EVIS LUCERA CV-260 colonoscopes (Olympus Co., Tokyo, Japan). We used a polyethylene glycol solution (Taejoon Pharm. Inc., Seoul, Republic of Korea) of 4 L for bowel cleansing. The presence of hemorrhoidal disease was defined as morphological presence of internal and/or external hemorrhoidal disease assessed by a colonoscopic exam. Internal hemorrhoidal disease was defined as the presence of an enlarged vein above the dentate line on retroflexion of the colonoscope. External hemorrhoidal disease was defined as the presence of an enlarged vein at or below the dentate line.

### Statistical analysis

The association between potential risk factors and the prevalence of hemorrhoidal disease was evaluated using logistic regression. We estimated the adjusted odds ratios (OR) and their 95% confidence intervals (CIs) for the prevalence of hemorrhoidal disease in models including age category (20–29, 30–39, 40–49, 50–59, and 60–70 years), sex, education (> 12 and ≤ 12 years), smoking (never, former, and current), alcohol intake (none, < 5 g/day, 5–20 g/day, and ≥ 20 g/day), physical activity (low, moderate, and high), BMI category (underweight, normal, overweight, and obese), and a history of diabetes, hypertension, and dyslipidemia. We also evaluated the independent association between different markers of adiposity (% fat mass, waist circumference, and central obesity), as well as the association between sex, parity, and mode of delivery and hemorrhoidal disease. In addition, we evaluated the association between each of the 13 gastrointestinal symptoms and hemorrhoidal disease in the fully adjusted model. Linear trends of the associations were also evaluated by using the median values for each category as a continuous variable in the regression models.

All statistical analyses were performed with Stata version 15.0 (StataCorp LP, College Station, TX, USA). All *P* values reported in the study are two-sided and *P* values < 0.05 were considered statistically significant.

### Ethics approval

The study was approved by the Institutional Review Board of the Kangbuk Samsung Hospital, which waived the requirement for informed consent because we used only de-identified information obtained as part of routine health screening exams.

## Supplementary Information

Supplementary Information.

## Data Availability

Upon request to the authors of the paper.

## Abbreviations

BMI: Body mass index
IPAQ-SF: International physical activity questionnaire short form
MET: Metabolic equivalent
