# Supplementary material for: Risk factors for hemorrhoidal disease among healthy young and middle-aged Korean adults
Source: Sci Rep. 2022 Jan 7;12:129. doi: 10.1038/s41598-021-03838-z (PMC8741788; doi:10.1038/s41598-021-03838-z)
Supplement: Supplementary file 1 — Supplementary Information. [file 41598_2021_3838_MOESM1_ESM.docx]

**Risk factors for hemorrhoidal disease among healthy young and middle-aged Korean adults**

**Short title: Risk factors for hemorrhoidal disease**

Yun Soo Hong, MD, MHS,^1*^ Kyung Uk Jung, MD,^2*^ Sanjay Rampal, MD, PhD,^3^ Di Zhao, PhD,^1^ Eliseo Guallar, MD, DrPH,^1,4^ Seungho Ryu, MD, PhD,^4-6^ Yoosoo Chang, MD, PhD,^4-6^ Hyung Ook Kim, MD, PhD,^2^ Hungdai Kim, MD, PhD,^2^ Ho-Kyung Chun, MD, PhD,^2^ Chong Il Sohn, MD, PhD,^5,7^ Hocheol Shin, MD, PhD, ^5,8^ Juhee Cho, PhD.^1,4,5^

^1^ Departments of Epidemiology and Medicine, and Welch Center for Prevention, Epidemiology, and Clinical Research, Johns Hopkins University Bloomberg School of Public Health. Baltimore, Maryland, USA.

^2^ Department of Surgery, Kangbuk Samsung Hospital, Sungkyunkwan University School of Medicine, Seoul, Korea.

^3^ Department of Social and Preventive Medicine, Julius Centre University of Malaya, Faculty of Medicine, University of Malaya, Kuala, Lumpur, Malaysia.

^4^ Department of Clinical Research Design and Evaluation, SAIHST, Sungkyunkwan University, Seoul, Republic of Korea.

^5^ Center for Cohort Studies, Total Healthcare Center, Kangbuk Samsung Hospital, Sungkyunkwan University School of Medicine, Seoul, Republic of Korea.

^6^ Department of Occupational and Environmental Medicine, Kangbuk Samsung Hospital, Sungkyunkwan University School of Medicine, Seoul, Republic of Korea.

^7^ Division of Gastroenterology, Department of Internal Medicine and Gastrointestinal Cancer Center, Kangbuk Samsung Hospital, Sungkyunkwan University School of Medicine, Seoul, Korea.

^8^ Department of Family Medicine, Kangbuk Samsung Hospital, Sungkyunkwan University School of Medicine, Seoul, South Korea.

^*^ Yun Soo Hong and Kyung Uk Jung equally contributed to this paper as co-first authors.

**Address for correspondence:** Juhee Cho, PhD, Department of Clinical Research Design & Evaluation, SAIHST, Sungkyunkwan University, 81 Irwon-ro, Gangnam-gu, Seoul, 06351, Korea. Tel: +(82) 02-3410-1448, Fax: +(82) 02-3410-6639; E-mail: jcho@skku.edu.

**Co-correspondence:** Ho-Kyung Chun, MD, PhD, Department of Surgery, Kangbuk Samsung Hospital, Sungkyunkwan University School of Medicine, 29 Saemunan-ro, Jongno-gu, Seoul, 03181, South Korea. Tel: +82-2-2001-2008, Fax: +82-2-2001-2016, E-mail: [hokyung.chun@samsung.com](mailto:hokyung.chun@samsung.com).

**Grant support:** None to declare.

**Conflict of interest (Disclosures):** There is no conflicts of interest to declare for all authors.

**Word count: 2,930**

**Abbreviations**

**BMI** Body mass index

**IPAQ-SF** International Physical Activity Questionnaire Short Form

**MET** Metabolic equivalent

**Appendix Table 1.** Characteristics of study participants by presence of hemorrhoidal disease.

|  | **Overall, n (%)** | **Hemorrhoidal disease, n (%)** | | ***P*** |
| --- | --- | --- | --- | --- |
|  | **(n = 194,620)** | **No**  **(n = 162,273)** | **Yes**  **(n = 32,347)** |  |
| **Age** |  |  |  | <0.001 |
| 20 to 29 | 11,474 (5.9) | 10,088 (6.2) | 1,386 (4.3) |  |
| 30 to 39 | 80,366 (41.3) | 69,214 (42.7) | 11,152 (34.5) |  |
| 40 to 49 | 63,853 (32.8) | 52,409 (32.3) | 11,444 (35.4) |  |
| 50 to 59 | 27,776 (14.3) | 21,868 (13.5) | 5,908 (18.3) |  |
| 60 to 70 | 11,151 (5.7) | 8,694 (5.4) | 2,457 (7.6) |  |
| **Men** | 125,435 (64.5) | 104,977 (64.7) | 20,458 (63.3) | < 0.001 |
| **Education >12 years** | 145,517 (74.8) | 121,919 (75.1) | 23,598 (73.0) | <0.001 |
| **Physical activity** |  |  |  | 0.001 |
| Low | 92,330 (47.4) | 76,937 (47.4) | 15,393 (47.6) |  |
| Moderate | 67,163 (34.5) | 56,259 (34.7) | 10,904 (33.7) |  |
| High | 32,736 (16.8) | 27,112 (16.7) | 5,624 (17.4) |  |
| **Smoking** |  |  |  | 0.01 |
| Never | 87,113 (44.8) | 72,804 (44.9) | 14,309 (44.2) |  |
| Former | 53,466 (27.5) | 44,383 (27.4) | 9,083 (28.1) |  |
| Current | 42,379 (21.8) | 35,423 (21.8) | 6,956 (21.5) |  |
| **Daily alcohol intake** |  |  |  | <0.001 |
| None | 20,447 (10.5) | 16,917 (10.4) | 3,530 (10.9) |  |
| <5 g/day | 56,053 (28.8) | 46,786 (28.8) | 9,267 (28.7) |  |
| 5 to <20 g/day | 60,666 (31.2) | 50,812 (31.3) | 9,854 (30.5) |  |
| ≥20 g/day | 45,816 (23.5) | 38,184 (23.5) | 7,632 (23.6) |  |
| **Body mass index** (kg/m^2^) | 23.8 (3.3) | 23.8 (3.3) | 23.8 (3.2) | 0.11 |
| **% Fat mass** (%) | 26.0 (6.6) | 25.9 (6.6) | 26.2 (6.6) | <0.001 |
| **Waist circumference** (cm) | 83.1 (9.4) | 83.1 (9.4) | 83.1 (9.2) | 0.47 |
| **Central obesity** | 51,425 (26.4) | 42,817 (26.4) | 8,608 (26.6) | 0.40 |
| **Diabetes** | 10,254 (5.3) | 8,336 (5.1) | 1,918 (5.9) | <0.001 |
| **Hypertension** | 27,826 (14.3) | 22,582 (13.9) | 5,244 (16.2) | <0.001 |
| **Dyslipidemia** | 8,604 (4.4) | 6,880 (4.2) | 1,724 (5.3) | <0.001 |

Values in the Table are number (%) or mean (SD).

**Appendix Table 2.** Association between participant characteristics and the prevalence of hemorrhoidal disease by sex and parity.

|  | **Men**  **(n = 125,435)** | **Women, Nulliparous**  **(n = 10,617)** | **Women, Parous**  **(n = 53,699)** | ***P* for interaction** |
| --- | --- | --- | --- | --- |
|  | **aOR^*^ (95% CI)** | **aOR^*^ (95% CI)** | **aOR^*^ (95% CI)** |  |
| **Age** |  |  |  | <0.001 |
| 20 to 29 | 1.00 (ref) | 1.00 (ref) | 1.00 (ref) |  |
| 30 to 39 | 1.20 (1.11, 1.29) | 1.26 (1.09, 1.46) | 1.12 (0.89, 1.40) |  |
| 40 to 49 | 1.63 (1.51, 1.75) | 1.57 (1.39, 2.02) | 1.32 (1.05, 1.65) |  |
| 50 to 59 | 1.99 (1.83, 2.15) | 1.90 (1.35, 2.69) | 1.60 (1.27, 2.01) |  |
| 60 to 70 | 2.17 (1.97, 2.39) | 1.42 (0.68, 3.00) | 1.53 (1.21, 1.95) |  |
| **Education** |  |  |  | 0.43 |
| 12+ years | 1.00 (ref) | 1.00 (ref) | 1.00 (ref) |  |
| ≤ 12 years | 1.00 (0.96, 1.05) | 1.09 (0.93, 1.28) | 1.03 (0.97, 1.08) |  |
| **Physical activity** |  |  |  | 0.93 |
| Low | 1.00 (ref) | 1.00 (ref) | 1.00 (ref) |  |
| Moderate | 0.97 (0.94, 1.00) | 0.95 (0.84, 1.08) | 0.98 (0.93, 1.03) |  |
| High | 0.98 (0.94, 1.02) | 0.97 (0.80, 1.16) | 1.01 (0.95, 1.08) |  |
| **Smoking status** |  |  |  | 0.30 |
| Never | 1.00 (ref) | 1.00 (ref) | 1.00 (ref) |  |
| Former | 1.05 (1.01, 1.09) | 0.97 (0.79, 1.19) | 1.09 (1.00, 1.19) |  |
| Current | 1.08 (1.03, 1.13) | 0.90 (0.68, 1.18) | 0.93 (0.78, 1.11) |  |
| **Daily alcohol intake** |  |  |  | 0.76 |
| None | 1.00 (ref) | 1.00 (ref) | 1.00 (ref) |  |
| <5 gm/day | 0.99 (0.92, 1.07) | 1.00 (0.82, 1.21) | 0.97 (0.92, 1.04) |  |
| 5 to <20 gm/day | 0.98 (0.91, 1.05) | 1.07 (0.88, 1.31) | 1.01 (0.94, 1.08) |  |
| ≥ 20gm/day | 0.98 (0.91, 1.05) | 1.08 (0.84, 1.39) | 1.01 (0.90, 1.14) |  |
| **Body mass index** |  |  |  | 0.07 |
| Underweight | 1.07 (0.92, 1.24) | 1.04 (0.88, 1.23) | 0.95 (0.87, 1.05) |  |
| Normal | 1.00 (ref) | 1.00 (ref) | 1.00 (ref) |  |
| Overweight | 1.01 (0.97, 1.05) | 1.08 (0.90, 1.29) | 1.10 (1.04, 1.17) |  |
| Obese | 0.97 (0.94, 1.01) | 0.97 (0.81, 1.16) | 1.10 (1.03, 1.17) |  |
| **Diabetes** |  |  |  | 0.90 |
| No | 1.00 (ref) | 1.00 (ref) | 1.00 (ref) |  |
| Yes | 0.93 (0.88, 0.98) | 0.88 (0.54, 1.44) | 1.03 (0.95, 1.13) |  |
| **Hypertension** |  |  |  | 0.12 |
| No | 1.00 (ref) | 1.00 (ref) | 1.00 (ref) |  |
| Yes | 1.04 (1.01, 1.07) | 0.95 (0.76, 1.18) | 1.07 (1.02, 1.12) |  |
| **Dyslipidemia** |  |  |  | 0.007 |
| No | 1.00 (ref) | 1.00 (ref) | 1.00 (ref) |  |
| Yes | 1.04 (0.98, 1.11) | 1.95 (1.18, 3.24) | 0.95 (0.85, 1.06) |  |

^*^ Adjusted for age (20–29, 30–39, 40–49, 50–59, and 60–70 years), sex, year of visit, center, education (≤12 and >12 years), physical activity (low, moderate, and high), smoking (never, former, and current), daily alcohol intake (none, <5 g/day, 5 to <20 g/day, and ≥20 g/day), BMI category (underweight, normal, overweight, and obese), and presence of diabetes, hypertension, and dyslipidemia.

**Appendix Table 3.** Association between alternative markers of adiposity and the prevalence of hemorrhoidal disease by sex and parity

|  | **Men**  **(n = 125,435)** | **Women, nulliparous**  **(n = 10,617)** | **Women, parous**  **(n = 53,699)** | ***P* for interaction** |
| --- | --- | --- | --- | --- |
|  | **aOR^*^ (95% CI)** | **aOR^*^ (95% CI)** | **aOR^*^ (95% CI)** |  |
| **% Fat mass** |  |  |  | 0.46 |
| Quintile 1 | 1.00 (ref) | 1.00 (ref) | 1.00 (ref) |  |
| Quintile 2 | 0.96 (0.92, 1.01) | 0.88 (0.74, 1.05) | 0.97 (0.90, 1.04) |  |
| Quintile 3 | 0.96 (0.91, 1.01) | 0.82 (0.69, 0.98) | 0.97 (0.90, 1.04) |  |
| Quintile 4 | 0.93 (0.89, 0.98) | 0.77 (0.64, 0.92) | 0.98 (0.91, 1.06) |  |
| Quintile 5 | 0.92 (0.88, 0.97) | 0.81 (0.67, 0.96) | 1.00 (0.93, 1.08) |  |
| **Waist circumference** |  |  |  | <0.001 |
| Quintile 1 | 1.00 (ref) | 1.00 (ref) | 1.00 (ref) |  |
| Quintile 2 | 1.02 (0.98, 1.07) | 1.08 (0.90, 1.28) | 1.07 (1.00, 1.16) |  |
| Quintile 3 | 0.99 (0.94, 1.04) | 0.91 (0.75, 1.09) | 1.08 (1.00, 1.16) |  |
| Quintile 4 | 1.01 (0.96, 1.06) | 0.92 (0.77, 1.11) | 1.09 (1.02, 1.18) |  |
| Quintile 5 | 0.94 (0.89, 0.99) | 1.01 (0.84, 1.22) | 1.16 (1.07, 1.25) |  |
| **Central obesity** |  |  |  | 0.005 |
| No | 1.00 (ref) | 1.00 (ref) | 1.00 (ref) |  |
| Yes | 0.96 (0.93, 1.00) | 1.01 (0.84, 1.22) | 1.08 (1.02, 1.15) |  |

^*^ Adjusted models included age (20–29, 30–39, 40–49, 50–59, and 60–70 years), sex, year of visit, center, education (≤12 and >12 years), physical activity (low, moderate, and high), smoking (never, former, and current), daily alcohol intake (none, <5 g/day, 5 to <20 g/day, and ≥20 g/day), and presence of diabetes, hypertension, and dyslipidemia, plus one of the following markers of excess adiposity: % fat mass % categorized in quintiles (separately for each group), waist circumference categorized in quintiles (separately for each group), and central obesity (no vs. yes).

**Appendix Table 4.** Association between self-reported gastrointestinal symptoms and the prevalence of hemorrhoidal disease by sex and parity.

|  | **Men**  **(n = 125,435)** | **Women, Nulliparous**  **(n = 10,617)** | **Women, Parous**  **(n = 53,699)** |
| --- | --- | --- | --- |
|  | **aOR^*^ (95% CI)** | **aOR^*^ (95% CI)** | **aOR^*^ (95% CI)** |
| **Difficulty swallowing** | 0.90 (0.72, 1.13) | 1.41 (0.71, 2.82) | 0.92 (0.71, 1.18) |
| **Acid regurgitation** | 1.02 (0.97, 1.08) | 1.13 (0.95, 1.34) | 0.97 (0.89, 1.04) |
| **Nausea and/or vomiting** | 0.99 (0.91, 1.08) | 0.94 (0.73, 1.20) | 0.93 (0.83, 1.05) |
| **Difficulty in digestion** | 0.97 (0.93, 1.01) | 1.02 (0.91, 1.15) | 0.96 (0.91, 1.01) |
| **Heartburn** | 1.01 (0.96, 1.06) | 1.02 (0.88, 1.17) | 0.97 (0.91, 1.03) |
| **Tarry stools** | 0.97 (0.88, 1.07) | 1.21 (0.87, 1.67) | 0.94 (0.77, 1.14) |
| **Fresh blood in stools** | 1.56 (1.47, 1.66) | 1.42 (1.14, 1.78) | 1.54 (1.35, 1.76) |
| **Frequent diarrhea** | 0.98 (0.94, 1.02) | 1.01 (0.88, 1.17) | 0.92 (0.85, 1.00) |
| **Constipation** | 1.07 (1.01, 1.14) | 1.10 (0.97, 1.24) | 1.14 (1.07, 1.20) |
| **Narrow caliber stools** | 1.05 (0.99, 1.11) | 1.12 (0.90, 1.39) | 1.06 (0.97, 1.16) |
| **Lump or mass in the abdomen** | 0.98 (0.84, 1.15) | 1.21 (0.91, 1.60) | 1.00 (0.97, 1.15) |
| **Frequent abdominal pain** | 1.00 (0.94, 1.07) | 1.08 (0.92, 1.27) | 1.03 (0.94, 1.12) |
| **Tenesmus** | 1.06 (1.02, 1.10) | 1.11 (0.97, 1.27) | 1.02 (0.96, 1.09) |

^*^ Adjusted for age (20–29, 30–39, 40–49, 50–59, and 60–70 years), sex, year of visit, center, education (≤12 and >12 years), physical activity (low, moderate, and high), smoking (never, former, and current), daily alcohol intake (none, <5 g/day, 5 to <20 g/day, and ≥20 g/day), BMI category (underweight, normal, overweight, and obese), and presence of diabetes, hypertension, and dyslipidemia.

**Appendix Table 5.** Associations of participant characteristics, markers of adiposity, and self-reported gastrointestinal symptoms with the prevalence of hemorrhoidal disease by parity and mode of delivery.

|  | **Women, Nulliparous**  **(n = 10,617)** | **Women, only cesarean section**  **(n = 7,072)** | **Women, ever given a natural birth**  **(n = 39,571)** |
| --- | --- | --- | --- |
|  | **aOR^*^ (95% CI)** | **aOR^*^ (95% CI)** | **aOR^*^ (95% CI)** |
| **Participant characteristics** | | | |
| **Age** |  |  |  |
| 20 to 29 | 1.00 (ref) | 1.00 (ref) | 1.00 (ref) |
| 30 to 39 | 1.21 (1.07, 1.36) | 1.60 (0.85, 3.03) | 1.05 (1.81, 1.37) |
| 40 to 49 | 1.42 (1.26, 1.62) | 1.96 (1.04, 3.71) | 1.21 (0.93, 1.57) |
| 50 to 59 | 1.76 (1.53, 2.01) | 2.40 (1.23, 4.70) | 1.52 (1.16, 1.98) |
| 60 to 70 | 1.64 (1.40, 1.91) | 1.59 (0.49, 5.11) | 1.42 (1.08, 1.88) |
| **Education** |  |  |  |
| 12+ years | 1.00 (ref) | 1.00 (ref) | 1.00 (ref) |
| ≤ 12 years | 1.00 (0.95, 1.06) | 1.00 (0.85, 1.16) | 0.99 (0.93, 1.06) |
| **Physical activity** |  |  |  |
| Low | 1.00 (ref) | 1.00 (ref) | 1.00 (ref) |
| Moderate | 0.97 (0.92, 1.02) | 0.95 (0.82, 1.09) | 0.98 (0.92, 1.04) |
| High | 1.01 (0.95, 1.08) | 1.00 (0.82, 1.20) | 1.03 (0.95, 1.11) |
| **Smoking status** |  |  |  |
| Never | 1.00 (ref) | 1.00 (ref) | 1.00 (ref) |
| Former | 1.05 (0.96, 1.15) | 1.04 (0.84, 1.30) | 1.08 (0.97, 1.21) |
| Current | 0.93 (0.79, 1.09) | 0.99 (0.64, 1.52) | 0.92 (0.74, 1.14) |
| **Daily alcohol intake** |  |  |  |
| None | 1.00 (ref) | 1.00 (ref) | 1.00 (ref) |
| <5 gm/day | 0.98 (0.92, 1.04) | 0.86 (0.72, 1.03) | 1.00 (0.93, 1.07) |
| 5 to <20 gm/day | 1.01 (0.94, 1.09) | 0.90 (0.73, 1.11) | 1.02 (0.93, 1.11) |
| ≥ 20gm/day | 1.04 (0.93, 1.17) | 0.81 (0.58, 1.13) | 1.10 (0.95, 1.26) |
| **Body mass index** |  |  |  |
| Underweight | 0.99 (0.91, 1.08) | 0.83 (0.62, 1.11) | 0.99 (0.89, 1.11) |
| Normal | 1.00 (ref) | 1.00 (ref) | 1.00 (ref) |
| Overweight | 1.12 (1.06, 1.19) | 1.15 (0.97, 1.35) | 1.13 (1.05, 1.21) |
| Obese | 1.08 (1.01, 1.15) | 1.24 (1.06, 1.45) | 1.07 (0.99, 1.15) |
| **Diabetes** |  |  |  |
| No | 1.00 (ref) | 1.00 (ref) | 1.00 (ref) |
| Yes | 1.02 (0.93, 1.12) | 1.06 (0.82, 1.37) | 1.02 (0.92, 1.13) |
| **Hypertension** |  |  |  |
| No | 1.00 (ref) | 1.00 (ref) | 1.00 (ref) |
| Yes | 1.07 (1.02, 1.12) | 0.96 (0.75, 1.22) | 1.09 (1.03, 1.15) |
| **Dyslipidemia** |  |  |  |
| No | 1.00 (ref) | 1.00 (ref) | 1.00 (ref) |
| Yes | 1.00 (0.89, 1.12) | 0.90 (0.55, 1.46) | 0.98 (0.87, 1.10) |
|  |  |  |  |
| **Alternative markers of adiposity** | | | |
| **% Fat mass** |  |  |  |
| Quintile 1 | 1.00 (ref) | 1.00 (ref) | 1.00 (ref) |
| Quintile 2 | 1.08 (0.95, 1.24) | 0.94 (0.63, 1.39) | 1.07 (0.91, 1.25) |
| Quintile 3 | 0.99 (0.88, 1.13) | 0.80 (0.55, 1.17) | 1.02 (0.88, 1.18) |
| Quintile 4 | 1.00 (0.89, 1.13) | 0.87 (0.61, 1.24) | 1.04 (0.90, 1.20) |
| Quintile 5 | 1.00 (0.89, 1.13) | 1.04 (0.74, 1.47) | 1.01 (0.88, 1.17) |
| **Waist circumference** |  |  |  |
| Quintile 1 | 1.00 (ref) | 1.00 (ref) | 1.00 (ref) |
| Quintile 2 | 1.04 (0.98, 1.10) | 1.04 (0.89, 1.21) | 1.05 (0.99, 1.13) |
| Quintile 3 | 1.09 (1.01, 1.16) | 1.16 (0.97, 1.40) | 1.08 (1.00, 1.17) |
| Quintile 4 | 1.10 (1.01, 1.20) | 1.15 (0.92, 1.44) | 1.10 (1.00, 1.22) |
| Quintile 5 | 1.07 (0.97, 1.18) | 1.47 (1.16, 1.86) | 1.04 (0.93, 1.16) |
| **Central obesity** |  |  |  |
| No | 1.00 (ref) | 1.00 (ref) | 1.00 (ref) |
| Yes | 1.08 (1.01, 1.15) | 1.28 (1.09, 1.49) | 1.05 (0.98, 1.13) |
|  |  |  |  |
| **Self-reported gastrointestinal symptoms** | | | |
| **Difficulty swallowing** | 0.94 (0.73, 1.21) | 1.41 (0.55, 3.60) | 0.86 (0.65, 1.14) |
| **Acid regurgitation** | 0.97 (0.90, 1.05) | 0.81 (0.64, 1.03) | 0.97 (0.88, 1.06) |
| **Nausea and/or vomiting** | 0.96 (0.85, 1.07) | 0.90 (0.64, 1.26) | 0.97 (0.85, 1.12) |
| **Difficulty in digestion** | 0.96 (0.91, 1.01) | 1.02 (0.88, 1.18) | 0.94 (0.88, 1.00) |
| **Heartburn** | 0.98 (0.92, 1.04) | 0.92 (0.78, 1.09) | 0.98 (0.92, 1.05) |
| **Tarry stools** | 0.97 (0.81, 1.16) | 0.95 (0.50, 1.80) | 0.89 (0.71, 1.11) |
| **Fresh blood in stools** | 1.52 (1.35, 1.72) | 1.11 (0.76, 1.63) | 1.68 (1.44, 1.97) |
| **Frequent diarrhea** | 0.94 (0.88, 1.02) | 1.01 (0.82, 1.24) | 0.90 (0.82, 1.00) |
| **Constipation** | 1.10 (1.05, 1.16) | 1.15 (0.99, 1.34) | 1.10 (1.03, 1.17) |
| **Narrow caliber stools** | 1.07 (0.98, 1.16) | 1.00 (0.76, 1.32) | 1.07 (0.96, 1.18) |
| **Lump or mass in the abdomen** | 1.00 (0.88, 1.15) | 1.17 (0.79, 1.75) | 0.93 (0.79, 1.10) |
| **Frequent abdominal pain** | 1.04 (0.96, 1.13) | 1.13 (0.90, 1.42) | 1.01 (0.91, 1.12) |
| **Tenesmus** | 1.06 (1.00, 1.12) | 1.10 (0.94, 1.30) | 1.03 (0.96, 1.11) |

^*^ Adjusted for age (20–29, 30–39, 40–49, 50–59, and 60–70 years), sex, year of visit, center, education (≤12 and >12 years), physical activity (low, moderate, and high), smoking (never, former, and current), daily alcohol intake (none, <5 g/day, 5 to <20 g/day, and ≥20 g/day), BMI category (underweight, normal, overweight, and obese), and presence of diabetes, hypertension, and dyslipidemia.
